# *Euphorbia hirta* Leaf Ethanol Extract Suppresses TNF-α/IFN-γ-Induced Inflammatory Response via Down-Regulating JNK or STAT1/3 Pathways in Human Keratinocytes

**DOI:** 10.3390/life12040589

**Published:** 2022-04-15

**Authors:** Tae-Young Gil, Sung-Chul Kang, Bo-Ram Jin, Hyo-Jin An

**Affiliations:** Department of Pharmacology, College of Korean Medicine, Sangji University, Wonju-si 26339, Gangwon-do, Korea; sophia14t@gmail.com (T.-Y.G.); schk315@daum.net (S.-C.K.); wlsqh92@gmail.com (B.-R.J.)

**Keywords:** *Euphorbia hirta*, keratinocytes, JNK, STAT1/3, inflammation

## Abstract

Skin inflammation may cause allergic diseases such as allergic rhinitis, asthma, and atopic dermatitis. *Euphorbia hirta* (*E. hirta*) is a member of the Euphorbiaceae family and is well-known for its anti-asthma effects. *E. hirta* has traditionally been used to treat respiratory ailments, dysentery, jaundice, and digestive problems. However, its effects on skin inflammation remain unclear. Here, we determined the effects of 70% ethanol extract of *E. hirta* leaves (ELE) in vitro using human keratinocyte HaCaT cells, which constitute most epidermal skin cells. We determined the inhibitory effects of ELE on the inflammation caused by tumor necrosis factor (TNF)-α/interferon (IFN)-γ in keratinocytes using ELISA, immunoblotting, and qRT-PCR assay. ELE was found to reduce the production and mRNA expression of pro-inflammatory cytokines such as TNF-α or interleukin-6 and the expression of various proteins, including signal transducers, activators of transcription 1/3, and mitogen-activated protein kinase. Expression levels of these proteins were found to be upregulated in the TNF-α/IFN-γ-stimulated condition and downregulated by ELE treatment. These results indicate that ELE protects HaCaT cells against TNF-α/IFN-γ-induced skin inflammation.

## 1. Introduction

The skin serves as the body’s primary barrier to the environment [1]. Each of the two main layers of skin, the epidermis and dermis, perform specific functions in maintaining homeostasis of the skin. In particular, the epidermal barrier reduces the absorption of chemicals, limits passive water loss from the body, and prevents microbial infection [2]. The skin thus acts as an identifiable receptor and effector organ for the management of cutaneous and systemic diseases [3]. Cutaneous inflammatory diseases, including atopic dermatitis (AD), are characterized by a typical progression to allergic rhinitis and asthma, which is also termed atopic march [4]. AD is a serious and incurable inflammatory skin disorder with a high relapse rate [5]. AD does not only cause pruritic and severe symptoms, but also reduces one’s quality of life and constitutes an economic burden [6]. To this end, the regulation of skin inflammation to maintain homeostasis may provide an alternative protective mechanism against systemic health disorders caused by the external environment.

Keratinocytes constitute around 90–95% of epidermal cells involved in the initiation and progression of immunological responses in the skin [7]. Here, we studied the effects of 70% ethanol extract (ELE) of *Euphorbia hirta* leaves on skin inflammation using immortalized human keratinocytes. When they are stimulated with pro-inflammatory cytokines, tumor necrosis factor (TNF)-α and interferon (IFN)-γ mixture (TNF-α/IFN-γ), keratinocytes are known to overexpress inflammatory factors [8]. The cells have also been widely used to investigate possible therapeutic agents against AD [9].

*Euphorbia hirta* (*E. hirta*), a member of the Euphorbiaceae family, has traditionally been used to treat gastrointestinal disorders, malaria, and inflammation [10,11]. *E. hirta* is also known as an asthma plant due to its therapeutic effects on respiratory diseases such as allergic asthma [12]. Although the effects of *E. hirta* on asthma and inflammatory diseases have been previously investigated in detail, its effect on skin allergic diseases such as AD remains unclear. Therefore, here, we examined the effect of ELE on TNF-α/IFN-γ-stimulated HaCaT keratinocytes, a cell line often used to study skin inflammation in vitro.

## 2. Materials and Methods

### 2.1. Chemicals and Reagents

Dulbecco’s modified Eagle’s medium (DMEM), inactivated fetal bovine serum (FBS), penicillin, and streptomycin were purchased from Life Technologies Inc. (Grand Island, NY, USA). Dimethyl sulfoxide (DMSO) was supplied by Junsei Chemical Co., Ltd. (Tokyo, Japan).

### 2.2. Cell Culture and ELE Treatment

HaCaT keratinocytes were cultured at 37 °C in DMEM supplemented with 10% inactivated FBS, penicillin (100 U/mL), and streptomycin (100 μg/mL) in a humidified atmosphere with 5% CO_2_. Cells were pre-treated with ELE at concentrations of 60, 120, and 240 μg/mL, and then stimulated using a mixture of TNF-α and IFN-γ (each 10 ng/mL) for the stipulated time, depending on the target markers.

### 2.3. Real-Time Reverse Transcription Polymerase Chain Reaction (RT-PCR)

Total RNA was isolated from HaCaT cells using an Easy Blue RNA extraction kit according to the manufacturer’s instructions. cDNA reverse transcription kits (Life Technologies, Grand Island, NY, USA) were used for synthesis. Reverse transcription was conducted using the GeneAmp PCR System 9700 (Applied Biosystems, Foster City, CA, USA) with SYBR Premix Ex Taq. The synthesized cDNAs were 200 base pairs in size. The StepOnePlus^®^ Real-Time PCR system (Applied Biosystems) was used for amplification with the SYBR Green PCR Master Mix. The results were expressed according to the comparative threshold cycle (Ct) method and were computed as the ratio of optical density to GAPDH. SYBR Premix Ex Taq was purchased from Takara Bio (Shiga, Japan). Oligonucleotide primers were supplied by Bioneer (Daejeon, Korea) (Table 1).

### 2.4. Western Blot Analysis

Protein extracts from HaCaT cells were prepared using the PRO-PREP™ protein extraction solution (Intron Biotechnology, Seoul, Korea). The protein concentration was determined using Bio-Rad assay reagent. Blots were visualized using enhanced specialized chemiluminescence (GE Healthcare Life Sciences, Chalfont, UK) and X-ray film (Agfa, Belgium). The details of the assay were as described in a previous study [13]. Primary antibodies were obtained from Cell Signaling Technology (Danvers, MA, USA) or Santa Cruz Biotechnology, Inc. (Santa Cruz, CA, USA) (Table 2).

### 2.5. Cytokine Analysis

Culture media were obtained approximately 24 h after treatment with ELE and stored at −70 °C. The levels of TNF-α and IL-6 were assessed using enzyme immunoassay (EIA) kits for humans (BD OptEIA^TM^, BD Science, San Jose, CA, USA) according to the manufacturer’s instructions.

### 2.6. Statistical Analyses

Data were expressed as the mean ± SD of three experiments. Comparisons among groups were performed using one-way analysis of variance (ANOVA), followed by Dunnett’s post hoc test. Statistical significance was set at *p* < 0.05.

## 3. Results

### 3.1. ELE Suppressed Production and mRNA Expression of Pro-Inflammatory Cytokines in HaCaT Keratinocytes

**Figure 1:**
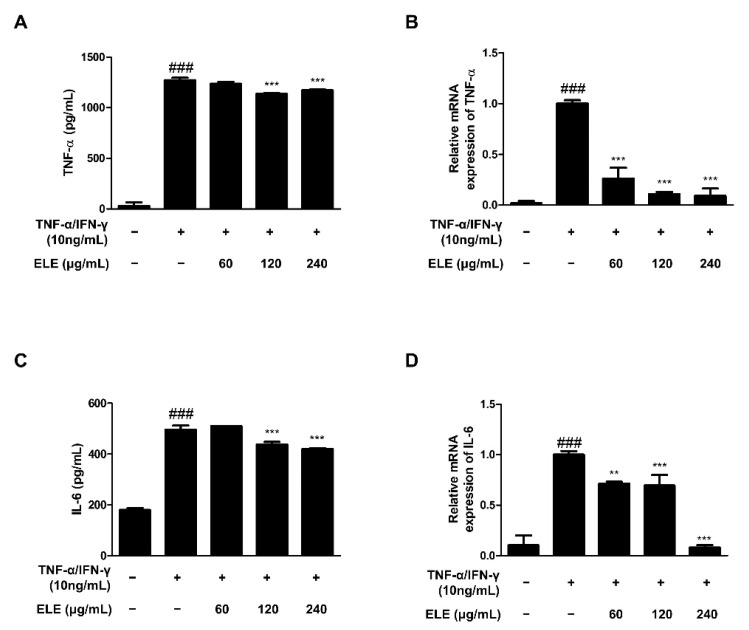
Effects of ELE treatment on inflammatory cytokines in HaCaT keratinocytes. Cytokine production and mRNA expression levels were evaluated. ELISA was used to determine the amount of (**A**) TNF-α and (**C**) IL-6 production. Cells were pre-treated with 60, 120, or 240 μg/mL of ELE for 1 h prior to additional treatment of TNF-α/IFN-γ and then incubated for 24 h. mRNA expression levels of (**B**) TNF-α and (**D**) IL-6 were determined by qRT-PCR. Data were presented as the mean ± SD; ** *p* < 0.01, *** *p* < 0.001 versus the only TNF-α/IFN-γ-treated group, ### *p* < 0.001 versus the control group; statistical significance of differences between the groups was evaluated by Dunnett’s post hoc test.

TNF-α is a representative molecule of pro-inflammatory cytokines [14]. TNF-α expression was found to be higher in the TNF-α/IFN-γ-treated group than that in the control group. The amount of TNF-α in the supernatant following TNF-α/IFN-γ treatment for 24 h was measured using an EIA kit (Figure 1A). The results indicated that the absorbance levels of the prepared standard and ELE-treated groups (120 and 240 μg/mL) were significantly reduced. We also used qRT-PCR to evaluate TNF-α mRNA expression levels following TNF-α/IFN-γ treatment for 6 h (Figure 1B). The results indicated a significant reduction with all ELE treatment concentrations in contrast to that of to the TNF-α/IFN-γ-treated group. IL-6 expression is also often increased with inflammation [15]. Indeed, we found downregulated IL-6 in ELE-treated groups at the protein and mRNA levels (Figure 1C,D). As with TNF-α production, notable downregulation was observed at 120 and 240 μg/mL ELE.

### 3.2. ELE Ameliorated mRNA Expression of Pro-Inflammatory Chemokines in HaCaT Cells

**Figure 2:**
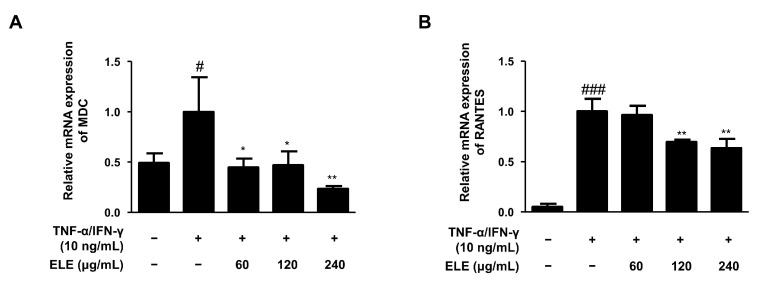
Effects of ELE treatment on inflammatory chemokines in HaCaT keratinocytes. mRNA expression levels of pro-inflammatory chemokines were determined. Cells were pre-treated with 60, 120, or 240 μg/mL of ELE for 1 h prior to TNF-α/IFN-γ addition, and incubated for 6 h. The mRNA expression levels of (**A**) MDC and (**B**) RANTES were determined by quantitative reverse transcription-polymerase chain reaction (qRT-PCR). Data were presented as the mean ± SD; * *p* < 0.05, ** *p* < 0.01 versus the only TNF-α/IFN-γ-treated group, # *p* < 0.05, ### *p* < 0.001 versus the control group; statistical significance of differences between the groups was evaluated by Dunnett’s post-hoc test.

The mRNA expression levels of two typical allergic chemokines, macrophage-derived chemokine (MDC, also known as CCL22) and regulated upon activation, and normal T cell expressed and secreted (RANTES, also known as CCL5), were evaluated [16]. The expression levels of these molecules were found to be downregulated by ELE treatment in human keratinocytes compared to those in the group stimulated only by TNF-α/IFN-γ (Figure 2).

### 3.3. ELE Inhibited Periostin Expression and Akt Phosphorylation in HaCaT Keratinocytes

**Figure 3:**
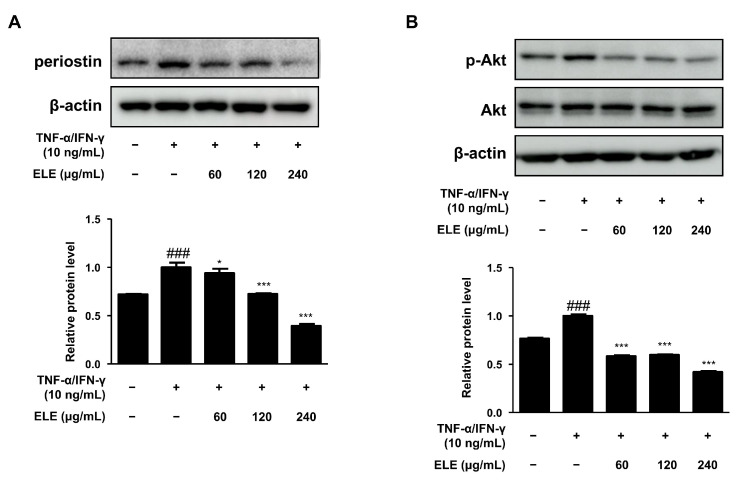
Effects of ELE treatment on inflammatory protein expression in HaCaT keratinocytes. HaCaT cells were treated with 60, 120, or 240 μg/mL of ELE for 1 h prior to an additional treatment of TNF-α/IFN-γ. Cells were then incubated for 15–20 min. The protein levels of (**A**) periostin and (**B**) phosphorylated Akt were determined by immunoblot analysis with specific antibodies. β-actin and Akt were used as internal controls. Densitometric analysis was performed using Image J software (version 1.50i). Values are presented as the mean ± SD of three independent experiments; * *p* < 0.05, *** *p* < 0.001 versus the only TNF-α/IFN-γ treated group, ### *p* < 0.001 versus the control group; statistical significance of differences between the groups was evaluated by Dunnett’s post hoc test.

Periostin is a critical mediator of amplified and persistent allergic reactions [17]. We also evaluated the expression of this protein. HaCaT cells were stimulated with TNF-α/IFN-γ for 15–20 min after pretreatment with ELE for 1 h. As a result, periostin levels were found to decrease at all ELE treatment concentrations, showing similar levels of normal control (Figure 3A). Akt activation is known to increase due to elevated inflammation or aged skin [18]. ELE treatment was found to suppress Akt phosphorylation following treatment with TNF-α/IFN-γ (Figure 3B).

### 3.4. ELE Ameliorated SEK1/MKK4-JNK Phosphorylation in HaCaT Keratinocytes

**Figure 4:**
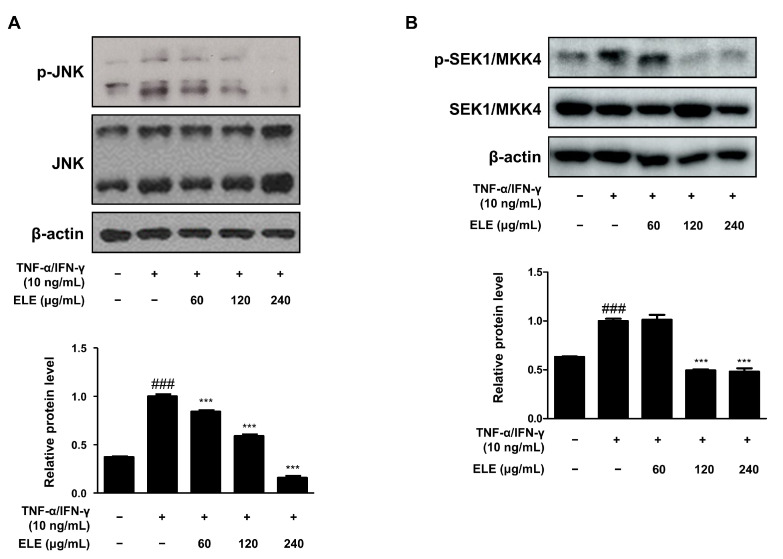
Effects of ELE treatment on SEK1/MKK4-JNK phosphorylation in HaCaT keratinocytes. HaCaT cells were treated with 60, 120, or 240 μg/mL of ELE for 1 h prior to an additional treatment with TNF-α/IFN-γ, and incubated for 10–15 min. The protein levels of phosphorylated (**A**) JNK and (**B**) SEK1/MKK4 were determined by immunoblot analysis with specific antibodies. JNK, SEK1/MKK4, and β-actin were used as internal controls. Densitometric analysis was conducted using Image J software (version 1.50i). Values are presented as the mean ± SD of three independent experiments; *** *p* < 0.001 versus the only TNF-α/IFN-γ treated group, ### *p* < 0.001 versus the control group; significances among the groups were evaluated by Dunnett’s post-hoc test.

The mitogen-activated protein kinase (MAPK) signaling pathway is involved in inflammation [19]. We thus determined the effect of ELE treatment on MAPKs as well. The cells were stimulated with TNF-α/IFN-γ for 10–15 min after pretreatment with ELE for 1 h. The JNK pathway was found to be downregulated upon ELE treatment (Figure 4A). Western blot assay results further showed a dose-dependent inhibitory effect of ELE treatment. Phosphorylation of SEK1/MKK4, a molecule involved in the JNK MAPK pathway, was also investigated at the protein level (Figure 4B). Treatment with ELE under TNF-α/IFN-γ-stimulated conditions was found to downregulate the phosphorylation tendency.

### 3.5. ELE Inhibited JAK2-STAT1/3 Activation in HaCaT Keratinocytes

**Figure 5:**
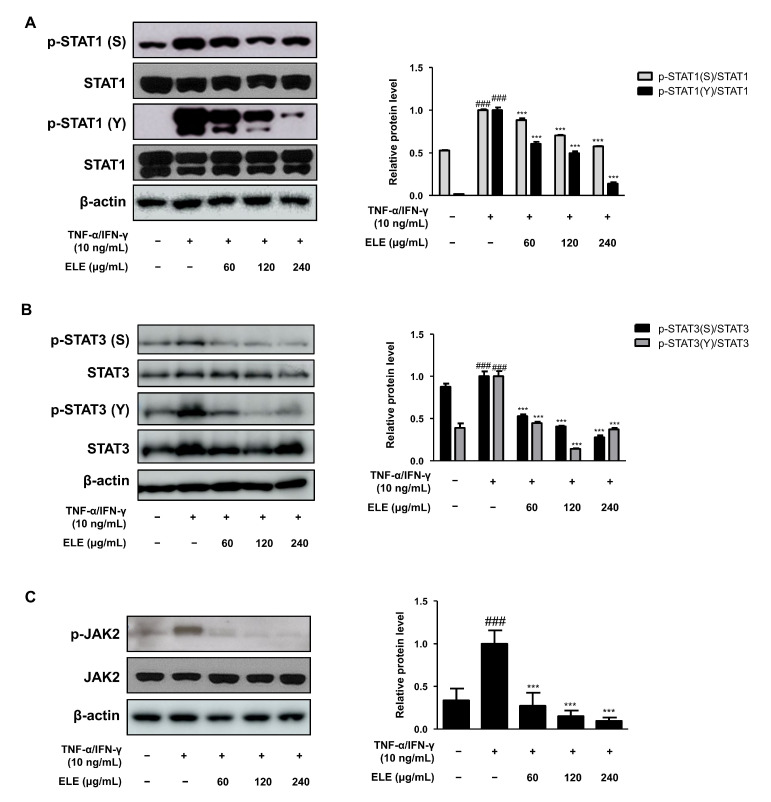
Effects of ELE treatment on JAK2-STAT1/3 phosphorylation in HaCaT keratinocytes. HaCaT cells were treated with 60, 120, or 240 μg/mL of ELE for 1 h prior to an additional treatment of TNF-α/IFN-γ and incubated for 10–15 min or 2 h. The protein levels of (**A**) STAT1, (**B**) STAT3, and (**C**) JAK2 phosphorylation were determined by immunoblot analysis with specific antibodies. STAT1, STAT3, JAK2, and β-actin were used as internal controls. Densitometric analysis was performed using Image J software (version 1.50i). Values are presented as the mean ± SD of three independent experiments; *** *p* < 0.001 versus the only TNF-α/IFN-γ treated group, ### *p* < 0.001 versus the control group; statistical significance of differences between the groups was evaluated by Dunnett’s post hoc test.

Another important signaling pathway involved in skin inflammation is Janus kinase (JAK)—signal transducer and activator of transcription (STAT) signaling [20,21]. We thus analyzed the level of STAT tyrosine (Y) and serine (S) phosphorylation (Figure 5A,B). ELE treatment caused the significant inhibition of phosphorylation of both STAT residues, serine, and tyrosine in TNF-α/IFN-γ-stimulated groups. JAK2 activation, which, in turn, phosphorylates STATs, was induced. ELE was found to suppress the activation of JAK2, STAT1, and STAT3 (Figure 5).

## 4. Discussion

AD is an allergic skin disease characterized by severe pruritus and inflammation due to T-helper type 2 (Th2)-mediated immune responses [5]. Numerous approaches to alleviate the symptoms of AD have been developed, yet no therapeutic agents with strong anti-AD effects have been found to date due to the complicated pathogenesis of the disease. Glucocorticoids or topical calcineurin inhibitors are frequently used to treat AD despite their many side effects such as skin atrophy [22]. Other treatment alternatives, such as topical phosphodiesterase 4 inhibitors, JAK-STAT inhibitors such as tofacitinib, or systemic biological treatments, are also favored in practice [23,24]. However, their high costs reduce accessibility to these treatments [25]. These limitations drive patients to seek safe treatments with high efficacy, few side effects, and low cost. A fundamental approach in this regard is the regulation of the inflammatory response. Here, we investigated the effects of ethanol extract of *E. hirta* leaves (ELE) on AD in human keratinocytes. The efficacy of *E. hirta* ELE in the treatment of allergic respiratory diseases has been previously investigated [12], yet its effects on another allergic disease, AD, have been investigated for the first time in this study.

Human epidermal keratinocytes (HaCaT), which are composed of keratinocytes, melanocytes, and Langerhans cells [26], were used in the present study. ELE treatment was not found to induce cytotoxicity at concentrations up to 1000 μg/mL and showed suppressive effects on pro-inflammatory cytokines such as IL-6 or TNF-α at the lower concentration (data not shown). Therefore, ELE concentrations of 60, 120, and 240 μg/mL were used in further experiments. Additionally, we observed the morphological changes between the normal group and TNF-α/IFN-γ-treated group under a microscope. The change was ameliorated in the high concentration of ELE (240 μg/mL).

The combination of TNF-α/IFN-γ stimulates keratinocytes to activate pro-inflammatory signaling pathways, which results in the production of inflammatory cytokines and chemokines [27,28]. Protein and mRNA expression levels of representative pro-inflammatory cytokines, including TNF-α and IL-6, were found to increase with inflammatory stimulation, and ELE treatment was found to suppress them (Figure 1). In particular, downregulation by ELE was significant in mRNA expression compared to the protein secretion using ELISA. It seemed to be caused by a later time point affecting the robust impact of ELE on protein expression with the experimental method [29,30]. In addition, mRNA expression levels of pro-inflammatory chemokines MDC/CCL22 and RANTES/CCL5 were found to decrease upon ELE treatment (Figure 2). MDC and RANTES are inflammatory chemokines that are mainly expressed in various immune cells such as lymphocytes, dendritic cells, and eosinophils [31]. Furthermore, the levels of chemokines in the serum and skin lesions of AD patients are also known to increase, suggesting that chemokines produced by keratinocytes are crucial for attracting inflammatory lymphocytes to the skin [32]. Inflammatory chemokines such as IFN-γ are generally known to induce inflammatory skin conditions such as psoriasis [33]. ELE treatment showed inhibitory effects on increased expression of pro-inflammatory chemokines caused by TNF-α and IFN-γ.

Other allergic responses due to periostin or the activation of Akt, which are pivotal mediators of escalated allergic reactions, have also been investigated [17] (Figure 3). Western blotting was used to evaluate protein expression levels. TNF-α/IFN-γ-stimulated keratinocytes showed increased levels of periostin, which were decreased upon ELE treatment in a dose-dependent manner. In addition, protein expression of phosphorylated Akt was evaluated under inflammatory conditions [34]. The inhibitory effect of ELE on inflammatory molecules is shown in Figure 3.

The effects of ELE treatment on inflammation-related signaling pathways, such as the JAK/STAT and MAPK pathways, were also investigated. STAT1/3 is involved in antiviral type 1 (Th1) responses [35] and regulates the elevated Th2 cell response and the maturation of B cells [36]. The JAK2-STAT1/3 signaling pathway was investigated in terms of protein expression levels (Figure 4 and Figure 5). Since JAK phosphorylates STAT, the dissociated STAT from the receptor forms homodimers or heterodimers via SH2-domain-phosphotyrosine interactions, translocating to promoters of target genes [37,38]. Here, ELE treatment was found to inhibit increased JAK2 activation by TNF-α/IFN-γ, and the phosphorylation of serine and tyrosine residues on STAT1/3 was decreased upon ELE treatment in a dose-dependent manner. In particular, the inhibitory effects of ELE on STAT1/3 tyrosine phosphorylation were significant compared to those in the TNF-α/IFN-γ-stimulated group. Typical Th1-type cytokines, TNF-α/IFN-γ mixture, caused HaCaT cells to activate the intracellular MAPK signaling pathway, and thereby induced the secretion of pro-inflammatory cytokines such as TNF-α or IL-6, mimicking AD-like responses [39,40]. Here, ELE treatment was found to downregulate the protein expression levels of phosphorylated SEK1/MKK4-JNK caused by TNF-α/IFN-γ in HaCaT keratinocytes (Figure 4 and Figure 5). JNK MAPK activation was found to be downregulated upon ELE treatment. This effect was considered to be related to an upstream pathway of JNK MAPK, SEK1/MKK4 phosphorylation, caused by TNF-α/IFN-γ. These observations suggest that ELE suppresses the activation of the intracellular pathways STAT1/3 or JNK MAPK by inhibiting JAK2 or SEK1/MKK4 phosphorylation, respectively. Hence, ELE was found to exert inhibitory effects on two types of inflammatory pathways.

At the same time, it is necessary to investigate the clear interaction mechanism of ELE through whether ELE showed the suppressive effects on the phosphorylation of STAT1/3 or JNK MAPK. The other experimental accesses could have facilitated our determination of the specific binding to a particular receptor. Otherwise, in vivo study of ELE could express the clear mechanism with pre-clinical development. It would be easier to figure out the mechanism of ELE with the results of chromatography demonstrating the contained compounds such as ellagic acid. With these points, we could perform further studies on ELE based on the effectiveness determined by the in vitro results.

In conclusion, we showed the anti-inflammatory effects of ELE on TNF-α/IFN-γ in keratinocytes via the downregulation of STAT1/3 signaling pathways. ELE treatment promoted the production and mRNA expression of pro-inflammatory cytokines in the human keratinocyte cell line, HaCaT. The results also indicated the inhibition of JNK MAPK or STAT1/3 signaling pathways.

## Figures and Tables

**Table 1:** Sequences of the RT-PCR primers.

Gene	Primer Sequences	
TNF-α (human)	Forward (5′-3′)	CGCTCCCAAGAAGACAG
	Reverse (5′-3′)	AGAGGCTGAGGAACAAGCAC
IL-6 (human)	Forward (5′-3′)	CCGGGAACGAAAGAGAAGCT
	Reverse (5′-3′)	AGGCGCTTGTGGAGAAGGA
MDC (human)	Forward (5′-3′)	AGGACAGAGCATGGATCGCCTACAGA
	Reverse (5′-3′)	TAATGGCAGGGAGGTAGGGCTCCTGA
RANTES (human)	Forward (5′-3′)	CCGCGGCAGCCCTCGCTGTCATCC
	Reverse (5′-3′)	CATCTCCAAAGAGTTGATGTACTCC
GAPDH (human)	Forward (5′-3′)	AATTCCATGGCACCGTCAAG
	Reverse (5′-3′)	ATCGCCCCACTTGATTTTGG

**Table 2:** List of primary antibodies.

Primary Antibody	Product No.	Primary Antibody	Product No.
p-JNK	cst #9251	JNK	cst #9252
p-SEK1/MKK4	cst #9156	SEK1/MKK4	cst #9152
p-STAT1 (Ser727)	cst #9177	p-STAT1 (Tyr701)	cst #9167
p-STAT3 (Ser727)	cst #9134	p-STAT3 (Tyr705)	cst #9145
STAT1	cst #9172	STAT3	sc-482
p-JAK2	cst #3776	JAK2	sc-390539
p-Akt	cst #9271	Akt	sc-8312
periostin	sc-398631	β-actin	sc-47778

## Data Availability

The data presented in this study are available on request by the corresponding author.
